# FFAR4 regulates cardiac oxylipin balance to promote inflammation resolution in HFpEF secondary to metabolic syndrome

**DOI:** 10.1016/j.jlr.2023.100374

**Published:** 2023-04-17

**Authors:** Naixin Zhang, Brian Harsch, Michael J. Zhang, Dylan J. Gyberg, Jackie A. Stevens, Brandon M. Wagner, Jenna Mendelson, Michael T. Patterson, Devin A. Orchard, Chastity L. Healy, Jesse W. Williams, DeWayne Townsend, Gregory C. Shearer, Katherine A. Murphy, Timothy D. O'Connell

**Affiliations:** 1 Department of Integrative Biology and Physiology, University of Minnesota, Minneapolis, MN, USA; 2 Department of Nutritional Sciences, The Pennsylvania State University, University Park, PA, USA; 3 Center for Immunology, University of Minnesota, Minneapolis, MN, USA

**Keywords:** omega-3 fatty acids, free fatty acid receptor 4 (Ffar4), heart failure preserved ejection fraction (HFpEF), metabolic syndrome, 18-hydroxyeicosapentaenoic acid, obesity, inflammation, lipidomics, phospholipase a2

## Abstract

Heart failure with preserved ejection fraction (HFpEF) is a complex clinical syndrome, but a predominant subset of HFpEF patients has metabolic syndrome (MetS). Mechanistically, systemic, nonresolving inflammation associated with MetS might drive HFpEF remodeling. Free fatty acid receptor 4 (Ffar4) is a GPCR for long-chain fatty acids that attenuates metabolic dysfunction and resolves inflammation. Therefore, we hypothesized that Ffar4 would attenuate remodeling in HFpEF secondary to MetS (HFpEF-MetS). To test this hypothesis, mice with systemic deletion of Ffar4 (Ffar4KO) were fed a high-fat/high-sucrose diet with L-NAME in their water to induce HFpEF-MetS. In male Ffar4KO mice, this HFpEF-MetS diet induced similar metabolic deficits but worsened diastolic function and microvascular rarefaction relative to WT mice. Conversely, in female Ffar4KO mice, the diet produced greater obesity but no worsened ventricular remodeling relative to WT mice. In Ffar4KO males, MetS altered the balance of inflammatory oxylipins systemically in HDL and in the heart, decreasing the eicosapentaenoic acid-derived, proresolving oxylipin 18-hydroxyeicosapentaenoic acid (18-HEPE), while increasing the arachidonic acid-derived, proinflammatory oxylipin 12-hydroxyeicosatetraenoic acid (12-HETE). This increased 12-HETE/18-HEPE ratio reflected a more proinflammatory state both systemically and in the heart in male Ffar4KO mice and was associated with increased macrophage numbers in the heart, which in turn correlated with worsened ventricular remodeling. In summary, our data suggest that Ffar4 controls the proinflammatory/proresolving oxylipin balance systemically and in the heart to resolve inflammation and attenuate HFpEF remodeling.

Heart failure with preserved ejection fraction (HFpEF) is a complex clinical syndrome in which patients present with symptomatic heart failure and preserved ejection fraction (≥50%) (1, 2). Currently in the US, HFpEF prevalence is over 3 million patients, exceeding heart failure with reduced ejection fraction (HFrEF) and its incidence is increasing annually (2). Unfortunately, therapies for HFrEF have shown no efficacy in HFpEF (1, 2, 3), notwithstanding the recent positive results of the sodium-glucose cotransporter 2 inhibitors to improve outcomes of patients with HFpEF (4, 5). HFpEF etiology is complex and heterogeneous, and patients tend to be older, female, and demonstrate significant phenotypic variation (1, 2). Clinically, attempts have been made to subdivide HFpEF patients based on etiology, and this has identified a predominant subset of HFpEF patients with comorbidities associated with metabolic syndrome (MetS) (6, 7, 8). Mechanistically, it has been proposed that systemic, nonresolving inflammation associated with MetS and other HFpEF comorbidities may promote HFpEF ventricular remodeling (1, 6, 7), which is characterized by *1*) vascular endothelial cell dysfunction, increased ROS production, and cell death; *2*) inflammation characterized by leukocyte (macrophage) infiltration, leading to activation of fibroblasts and interstitial fibrosis; and *3*) decreased cardiac myocyte nitric oxide (NO) production and dysfunction, all contributing to increased passive stiffness, impaired filling, and exercise intolerance (9, 10, 11, 12).

Free fatty acid receptor 4 (Ffar4, GPR120) is a G-protein coupled receptor (GPCR) for endogenous medium- and long-chain saturated, monounsaturated, and polyunsaturated fatty acids (SFA, MUFA, and PUFA) (13). This includes, but is not limited to, the cardioprotective ω3-PUFAs, eicosapentaenoic acid (EPA) and docosahexaenoic acid (DHA) (14). Ffar4 is expressed in several tissues with similar expression patterns in mouse and human (15) and high levels of expression in lung, brain, and gastrointestinal-tract but lower levels in other tissues including pancreas, small intestine, adipose, taste buds, muscle, heart, liver, and macrophages (15, 16, 17).

Ffar4 is a Gq-coupled receptor that activates both Gq- and βarrestin2-mediated pathways and is proposed to attenuate metabolic dysfunction and resolve inflammation (18, 19). However, the role of Ffar4 in regulating metabolic function remains somewhat controversial. In general, previous studies in mouse models indicate that in response to a metabolic challenge with high-fat diet (HFD), loss of Ffar4 worsens metabolic disease, with evidence of insulin resistance, glucose intolerance, adipose dysfunction, and fatty liver but with little or no effect on weight gain (20, 21, 22, 23). In humans, Ffar4 expression is increased in adipose from obese individuals, and in a European cohort, the *FFAR4* R270H inactivating polymorphism is associated with morbid obesity (21). However, other studies have refuted these findings, suggesting loss of Ffar4 in mice does not worsen metabolic disease (24, 25), and in a separate Danish cohort, there was no association between R270H and obesity (26). On the other hand, activation of Ffar4 with synthetic ligands including compound A, TUG-891, or compound 34 generally improves metabolic dysfunction and insulin resistance (27, 28, 29).

As noted, Ffar4 also attenuates inflammation in a variety of settings. In macrophages, Ffar4 activation inhibits NFkB signaling and subsequent production of the inflammatory cytokines interleukin-6 and TNFα, rendering these macrophages less inflammatory and attenuating adipocyte dysfunction in obesity (23, 28). Additionally, Ffar4-mediated activation of cytoplasmic phospholipase A2α (cPLA2α) in macrophages induces the production of oxidatively modified FAs, or oxylipins, that attenuate proinflammatory signaling (30, 31, 32).

Although we previously detected Ffar4 expression in both cardiac myocytes and fibroblasts (33), little was known about the physiologic function of Ffar4 in the heart until we recently demonstrated that Ffar4 is cardioprotective in a model of pathologic pressure overload (transverse aortic constriction [TAC]). Although we found that mice with systemic deletion of Ffar4 (Ffar4 knockout mice, Ffar4KO) had no obvious phenotype at baseline, loss of Ffar4 amplified TAC-induced hypertrophy without excessive fibrosis and worsened TAC-induced systolic and diastolic dysfunction in males but not females (34). In adult cardiac myocytes, we observed that Ffar4-cPLA2α signaling specifically and uniquely induced the production of the EPA-derived, cardioprotective, proresolving oxylipin 18-hydroxyeicosapentaenoic acid (18-HEPE), which attenuated cardiac myocyte death induced by oxidative stress (34). In humans, we found that *FFAR4* R270H was associated with left ventricular hypertrophy and dilation in a cohort of 7,140 genotyped subjects who had a clinically indicated echocardiogram (34).

The beneficial effects of Ffar4 on metabolism and inflammation (21, 23), the high incidence of MetS in HFpEF patients (8), and the recently described cardioprotective effects of Ffar4 (34) led us to hypothesize that loss of Ffar4 would worsen ventricular remodeling in a mouse model of HFpEF secondary to MetS (HFpEF-MetS). To test this hypothesis, we modified the 2-hit model developed by Schiattarella *et al.* (35), by using a 42% high-fat/30% high-sucrose Western diet, which more closely resembles human dietary patterns (36), to induce obesity and Type 2 diabetes along with the NO synthase-inhibitor Nω-Nitro-L-arginine methyl ester (L-NAME) hydrochloride in the drinking water to induce hypertension. Here, we report that in male Ffar4KO mice, this HFpEF-MetS diet induced similar metabolic deficits but worsened diastolic function and microvascular rarefaction relative to WT mice. Conversely, female Ffar4KO mice responded with greater obesity but no worsening of HFpEF. In male Ffar4KO mice, MetS altered the balance of inflammatory oxylipins both systemically in HDL and in the heart, decreasing 18-HEPE levels, while increasing levels of the arachidonic acid (AA)-derived, proinflammatory oxylipin 12-hydroxyeicosatetraenoic acid (12-HETE). This increased 12-HETE/18-HEPE ratio potentially reflects a more proinflammatory state systemically in the heart and was associated with increased CD64^+^ macrophages in the heart, which in turn correlated with worsened ventricular remodeling in male Ffar4KO hearts. In a broader context, our data suggest that Ffar4 prevents the negative impact of MetS in the heart.

## Materials and methods

### Mice

Ffar4KO mice were generated from cryopreserved sperm from C57Bl/6N-*Ffar4*^tm1(KOMP)Vlcg^ (Design ID: 15078; Project ID: VG15078) purchased from The Mutant Mouse Resource and Research Centers, UC-Davis (Davis, CA) as previously described (34). Mice are congenic with continuous backcrossing into C57Bl6/J mice from Jackson Labs. Heterozygous mice from these backcrosses are bred to produce WT and Ffar4KO mice, homozygous progeny is subsequently bred to produce WT and Ffar4KO mice, as two separate lines. All breeders (from het-het crosses or WT or Ffar4KO crosses) are replaced every 4–6 months.

At 8 weeks of age, male and female, WT and Ffar4KO mice were randomized to be fed a control diet (DYET #104607, Dyets Inc., Bethlehem, PA) or a diet designed to induce HFpEF-MetS for 20 weeks. Specifically, this HFpEF-MetS diet consisted of the combination of a 42% fat and 30% sucrose chow (DYET #104608, Dyets Inc) and L-nitroarginine methyl ester (L-NAME, (1 mg/ml, Cat #N5751, Sigma Chemical, St Louis, MO) in the drinking water. The compositions of the high-fat/high-sucrose diet and the specifically designed control diet are included in supplemental Table 1A and B. Mice were maintained on a 12-h light/dark cycle at 25°C with ad libitum access to food and water. For all experimental analyses, data collection was done with investigator blinded to genotype and treatment.

The HFpEF-MetS diet was designed to induce metabolic syndrome: obesity, hypertension, Type 2 diabetes, increased levels of plasma triglycerides (TGs), and decreased levels of plasma HDL. As such, exclusion criteria included failure to induce obesity (adiposity index (AI) < 0.25) and/or hypertension (systolic blood pressure < 120 mmHg) after 20 weeks on the HFpEF-MetS diet. Of the 86 WT and 93 Ffar4KO mice randomized to the HFpEF-MetS diet, 3 WT male, and 7 WT female were excluded, whereas 5 Ffar4KO male and 2 Ffar4KO female were excluded based on these exclusion criteria.

### Body weight and body composition

Body weights were obtained weekly for all mice for 20 weeks. To measure body composition, EchoMRI scans (EchoMRI, Houston, TX) were performed on all mice after 20 weeks on diet to record fat mass, lean mass, and free water. The AI was calculated by the equation: AI = fat mass/lean mass.

### Blood pressure

Blood pressure (BP), including mean arterial pressure (MAP), systolic pressure (SP), and diastolic pressure (DP), was measured in all mice after 20 weeks using the CODA noninvasive tail-cuff BP system (Kent Scientific, Torrington, CT). The procedure involved 2 days of acclimation, followed by measurements of BP on two subsequent days to determine the average BP. For acclimation, mice were placed in cylindrical restrainers for 15 min each day prior to the test. Prior to measurements of BP, the Occlusion (O)-cuff and volume/pressure recording (VPR)-cuff were tested for patency, per manufacturer’s instructions. For measurements of BP, mice were placed into cylindrical restrainers, with the tail left outside to attach the O-cuff and VPR cuff, and placed on a warming plate to acclimate and allow the tail temperature reached 35°C. Once at temperature, the O-cuff was placed at the base of the tail followed by the VPR-cuff. Tail temperature was closely monitored throughout the experiment and maintained between 35°C and 36°C. During each session, 25 BP measurement cycles were recorded, with the first 3 or 4 cycles considered acclimation cycles.

### Intraperitoneal glucose tolerance test

Blood glucose was sequentially measured at 0, 1, 15, 30, 60, and 120 min following an intraperitoneal glucose challenge in all mice after 20 weeks. Briefly, after an overnight fast (14 h), fasting glucose level was measured from tail blood using Quintet AC® Blood Glucose Monitor (McKesson, Irving, TX). Mice were then injected i.p. with D-glucose at 2 g/kg fasted body weight (#D16-500, Thermo Fisher Scientific, Waltham, MA) and glucose was again measured from tail blood at the indicated time points.

### Triglyceride and high-density lipoprotein

Blood was collected from the retro-orbital plexus into EDTA tubes from all mice after 20 weeks as previously described (34), total HDL-C and TG were measured by colorimetric assays. HDL-C was measured using a HDL-C E kit (Wako Laboratory Chemicals; Richmond, VA) and TG measured using an Infinity reagent colorimetric kit (Thermo Fisher Scientific; Waltham, MA).

### Cardiac function

Cardiac function was measured by echocardiography in all mice after 20 weeks using the Vevo 2100 system (FujiFilm VisualSonics Inc. Toronto, ON, Canada) with a MS400 transducer. For all measurements, mice were anesthetized with isoflurane, gently restrained in the supine position on the prewarmed monitoring pad, and echocardiographic images were captured as mice were recovering from anesthesia to achieve a target heart rate (HR) of 450–500 bpm. Isoflurane was maintained at 2–5% and adjusted accordingly in order to maintain a HR of 400–500 bpm. Parasternal long axis M-mode images of the left ventricle were captured to measure left ventricular parameters including: left ventricular posterior wall thicknesses (LVPW;s: systolic left ventricular posterior wall; LVPW;d: diastolic left ventricular posterior wall), left ventricular internal diameters (LVID;s: systolic left ventricular internal diameter; LVID;d: diastolic left ventricular internal diameter), left ventricular volumes (end systolic volume (ESV), ((7.0/(2.4 + LVID;s))∗LVID;s^3^); end diastolic volume (EDV), ((7.0/(2.4 + LVID;d))∗LVID;d^3^)), fractional shortening (FS: 100∗((LVID;d – LVID;s)/LVID;d)), ejection fraction (EF: 100∗((EDV – ESV)/EDV)), stroke volume (SV: EDV – ESV), and cardiac output (CO: SV∗HR). Pulsed-wave Doppler images of the apical four-chamber view and Tissue Doppler images at the level of mitral valve were captured to measure diastolic function, including peak velocity of mitral flow from left ventricular relaxation in early diastole (E wave), peak velocity of mitral flow from left ventricular relaxation in late diastole (A wave), and peak early diastolic mitral annular velocity (e’). Parasternal short axis Pulse-wave Doppler images were captured at the level of aortic valve to measure parameters of pulmonary artery flow including pulmonary acceleration time and pulmonary ejection time.

#### Strain analysis

Global longitudinal strain (GLS) and reverse longitudinal peak strain rate were measured using the speckle-tracking based imaging analysis in the VisualSonics Vevo LAB software version 5.5.0 (Toronto, Canada). Briefly, cine loops of the B-mode in parasternal long axis view were captured. Three consecutive cardiac cycles were chosen to manually draw the endocardial wall border and trace the movement of endocardium over time.

#### Langendorff-perfused hearts

Following 20 weeks of experimental treatment, mice were injected with 100 U of heparin and a barbiturate overdose. Hearts were rapidly harvested and placed in ice cold Krebs physiological salt solution. The aorta was cannulated and the heart was then perfused with warm oxygenated Krebs solution as previously described (37). A balloon was placed in the left ventricle to monitor ventricular pressures, oxygen probes monitored the oxygen concentrations in the perfusate and effluent, and a Transonic ultrasonic flow probe. The DP was set to 10 mmHg and the heart was given a 20-min equilibration period before the protocol began. After collection of baseline contractile data, the DP-volume relationship was determined. This was accomplished by removing volume from the ventricular balloon until the systolic pulse was no longer detected, then 5 μl of fluid was added back every minute until the DP reached 30 mmHg. After this protocol the DP was again set to 10 mmHg and the heart allowed to recover for 10 min. After this second equilibration period, 500 nM (−)-isoproterenol (Sigma) was infused directly into the coronary arteries. Maximum contractile response was measured within 5 min of the start of the infusion.

### Tissue histology

Ventricular fibrosis and microvascular density were measured in hearts harvested from mice after 20 weeks. Hearts were injected with 200 μl cold PBS with 60 mM KCl to arrest in diastole, excised, weighed, and subsequently perfused with PBS with 60 mM KCl to clear the heart of blood at 4 ml/min rate for 4 min. Atria were trimmed before hearts were flash-frozen in isopentane over liquid N_2_ in OCT embedding medium. Ten-micron cryosections at the midventricular level were cut and mounted at −25°C.

#### Fibrosis

Cryosections were stained in 0.1% solution of Sirius red (direct red 80, Sigma-Aldrich, St Louis, MO) and fast green (Sigma-Aldrich) in 1.2% picric acid (Ricca Chemical Company, Arlington, TX) (20 min, 25°C). To quantify LV fibrotic area, sections were imaged at 10X using a BZ-X800 fluorescent microscope (Keyence, Itasca, IL). Using NIH FIJI software, the color balance of all images was corrected before quantification. Using the color threshold function, picrosirius red positively stained area (fibrotic area) and whole ventricle area (right and left) were defined and fibrotic area/ventricular area was calculated.

#### Microvascular (capillary) density

Cryosections were stained with isolectin GS-IB4 (Alexa Fluor™ 594 conjugated; Thermo Fisher Scientific # I21413) at 20 μg/ml (2 h, 25°C). and counterstained with 4',6-diamidino-2-phenylindole at 1 μg/ml (5 min, 25°C). To quantify capillary density, sections were imaged at 20X using a Keyence BZ-X800 fluorescent microscope. Using NIH FIJI software, images were converted to 8 bit images, and following the application of the threshold function, images were further converted into bicolor mode. Subsequently, 7–9 regions of interests in a section with a total area between 400,000 and 600,000 μm^2^ near the endocardium were randomly selected for analysis. To quantify capillary density, we used an automated quantification algorithm for particle counting (stained capillary endothelium) in which we considered that the diameter of a capillary to be larger than 3 μm, and thus we set the particle size threshold to greater than 7.1 μm^2^. Using this algorithm, average capillary density of the total area was calculated in two representative sections from each heart.

#### Macrophage staining

Cryosections were stained with primary antibodies to detect CD64 (R&D Systems, Minneapolis, MN #AF2074) and subsequently stained with secondary antibodies conjugated to Alexa fluor 568 (donkey anti-goat, Thermo Fisher Scientific, Waltham, MA #A11057). To quantify macrophage density, sections were imaged at 20X using a BZ-X800 fluorescent microscope. Using NIH FIJI software, image contrast was adjusted to better visualize the positive staining. Following conversion to 8 bit images and application of the threshold function, images were further converted into bicolor mode. Subsequently, 7–9 regions of interests with a total area between 600,000 and 2,000,000 μm^2^ near the endocardium were randomly selected to calculate the number of CD64^+^ macrophages using an automated quantification algorithm to count particles (positively stained cells). Using this algorithm, macrophage density of the total area was calculated from each heart and was averaged within each treatment group.

### Hydroxyproline content

A hydroxyproline assay kit (Sigma, Burlington, MA #MAK008) was used to quantify total collagen content in the heart. After 20 weeks, hearts were harvested and a portion of the ventricle, ∼20 mg, was frozen and homogenized in ultrapure water. The ventricular tissue was hydrolyzed in 12 M HCl, and after 3 h at 120°C in pressure-tight capped vials, samples were centrifuged at 10,000 *g* for 3 min at room temperature. Subsequently, 10 μl (HFpEF samples) or 40 μl (control samples) of supernatant was transferred to the 96-well plate and dried overnight at 60°C. After drying, the Chloramine T/Oxidation Buffer Mixture provided with the hydroxyproline kit was added to both the sample and standard curve wells and incubated at room temperature. After 5 min, diluted 4-(dimethylamino)benzaldhyde reagent provided with the kit was added to all wells and incubated at 60°C for 90 min. Finally, absorbance was read at 560 nm using a Synergy H1 plate reader (Agilent Technologies, Santa Clara, CA). Hydroxyproline content was calculated using the standard curve and was normalized to tissue weight.

### Cardiac and HDL oxylipin content

Cardiac tissue oxylipins were taken from the apex of mouse hearts. Each tissue sample weight was measured (approximately 50 mg) and recorded and subjected to homogenization followed by oxylipin extraction. Plasma lipoproteins were separated by fast protein liquid chromatography followed by measurement of esterified oxylipins in HDL as previously described (34). HDL and homogenized cardiac tissue samples were spiked with BHT/EDTA (0.2 mg/ml), six deuterated octadecanoid, and eicosanoid surrogates (20 μl of 1000 nM concentration with final concentration of 50 nM after reconstitution) and subjected to liquid-liquid extraction to isolate lipid content. Samples were then hydrolyzed in 0.1 M methanolic sodium hydroxide to release ester-linked oxylipins and subjected to solid phase extraction using Chromabond HLB sorbent columns (Machery Nagel, Duren, Germany). Oxylipins were eluted with 0.5 ml of methanol with 0.1% acetic acid and 1 ml of ethyl acetate and dried under nitrogen stream and reconstituted in 200 ml methanol acetonitrile (1:1) with 100 nM of 1-cyclohexyluriedo-3-dodecanoic acid used as internal standard.

Samples were analyzed by liquid chromatography using a Waters Acquity UPLC coupled to Waters Xevo triple quadrupole mass spectrometer equipped with electrospray ionization source (Waters, Milford, MA). Five milliliters of the extract was injected, and separation was performed using a CORTECS UPLC C18 2.1 × 100 mm with 1.6 μM particle size column. Flow rate was set at 500 ml/min and consisted of a gradient run using water with 0.1% acetic acid (Solvent A) and acetonitrile isopropanol, 90:10 (Solvent B) for 15 min (25–95% of Solvent B was set from 0–11 min and held at 100% Solvent B from 11–13 min. The system was then re-equilibrated and conditioned at 25% Solvent B from 13–15 min). Electrospray ionization operated in negative ion mode with capillary set at 2.7 kV, desolvation temperature set at 600°C, and source temp set to 150°C. Optimal oxylipin multiple reaction monitoring transitions were previously identified by direct injection of pure standards onto the mass spectrometer and using cone voltage and collision energy ramps to optimize detection and most prevalent daughter fragments. Calibration curves were generated prior to each run using standards for each oxylipin. Peak detection and integrations were achieved through Target Lynx (Waters, Milford, MA) and each peak was inspected for accuracy and corrected when needed.

### RNA isolation and qPCR

RNA was isolated from the apex of the heart using the RNeasy Fibrous Tissue Mini Kit (Qiagen, Germantown MD #74704) after 20 weeks. RNA concentration was determined by NanoDrop Spectrophotometer (Thermo Fisher Scientific, Waltham, MA) and cDNA was synthesized by reverse transcription using the qScript cDNA SuperMix Kit (Quantabio, Beverly, MA #95047-100). Target gene expression was quantified by qRT-PCR using the Bio-Rad (Hercules, CA) iTaq Universal SYBR Green SuperMix (#1725120) and CFX96 Real-Time System.

Primer sequences:

**Ffar4 fwd** CGGCGGGGACCAGGAAAT.

**Ffar4 rvs** GTCTTGTTGGGACACTCGGA.

**Gpr31 fwd** CCACCAGTCTGCCATTCTTTG,

**Gpr31 rvs**ACTGTCGTCAGGAAGGCTACT.

**Cmklr1 fwd** ATGGAGTACGACGCTTACAACG.

**Cmklr1 rvs**GGTGGCGATGACAATCACCA.

### Statistical analysis

Cardiac phenotyping was analyzed using two-way ANOVA with a Tukey’s posttest using Prism 9.0 (GraphPad Software Inc, San Diego, CA). Where specified, principal components analysis was used for dimension reduction of oxylipin matrices on log-transformed, standardized concentrations. Mixed models were used to account for within mouse differences in oxylipin concentrations. Statistical significance was set at 0.05; Tukey’s test was used to test for specified posthoc differences using JMP version 13.2.1.

### Study approvals

#### Animal

All procedures on animals conformed to the NIH Guide for the Care and Use of Laboratory Animals and were reviewed and approved by the Institutional Animal Care and Use Committee at the University of Minnesota.

#### Additional compliance statements

After 20 weeks, mice were anesthetized with 3% isoflurane, verified by toe-pinch, followed by removal of the heart in accordance with recommendations from the American Veterinary Medical Association. Finally, all the data underlying this article are available in the article and in its online supplementary material.

## Results

### Loss of Ffar4 had no effect on the development of MetS in male mice

**Fig. 1:**
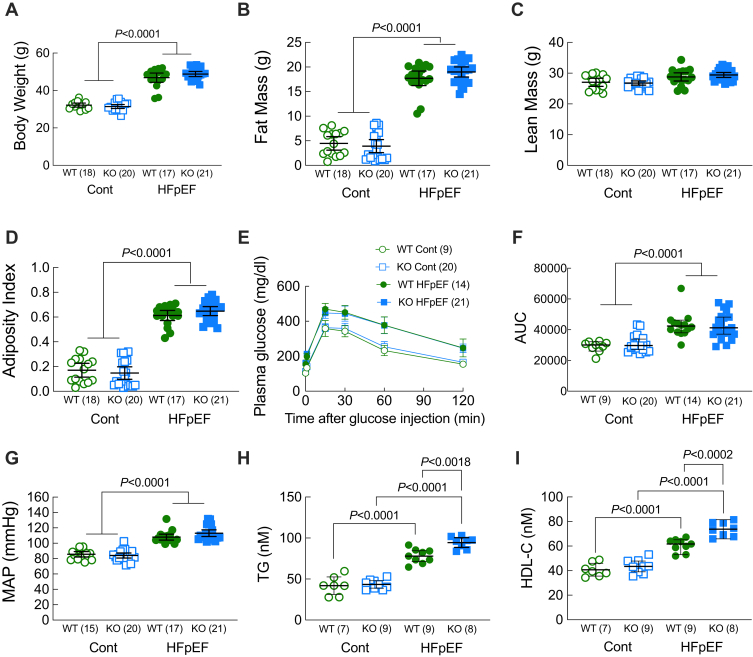
Male WT (green) and Ffar4KO (KO, blue) mice were fed a control diet (Cont., open symbols) or the combination of a high-fat (42%)/high-sucrose (30%) diet and L-NAME (1 mg/ml) in the drinking water (HFpEF, closed symbols) for 20 weeks. After 20 weeks; (A) Body weight was recorded, and body composition, including: (B) Fat Mass and (C) Lean Mass were determined by EchoMRI; and (D) Adiposity Index (fat mass/lean mass) was calculated. (E) Intraperitoneal glucose tolerance test (IPGTT) and (F) calculation of AUC. (G) Mean Arterial Pressure (MAP) measured by tail-cuff. (H) Triglyceride (TG) levels and (I) HDL-C levels were measured from plasma. Data (A–I) are presented as mean ± 95% confidence interval and were analyzed by two-way ANOVA with Tukey's multiple comparison test. Ffar4, free fatty acid receptor 4; HFpEF, heart failure with preserved ejection fraction; L-NAME, L-nitroarginine methyl ester.

To test our hypothesis that loss of Ffar4 would worsen HFpEF-MetS, we developed a dietary intervention designed to induce HFpEF secondary to MetS, by adapting the recently described 2-hit model (35). Here, mice were fed a high-fat/high-sucrose diet (42% fat/30% sucrose, less fat, and more sucrose than (35)) with L-NAME (1 mg/ml in the drinking water) designed to induce MetS (HFpEF-MetS diet). At 8 weeks of age, male and female, WT and Ffar4KO mice were randomized to the control diet (Cont diet: a standard diet designed as a control, no L-NAME) or the high-fat/high-sucrose/L-NAME diet (HFpEF-MetS diet) (Diet composition: supplemental Table 1A and B, Summary data: supplemental Table 2A). After 20 weeks, the HFpEF-MetS diet induced similar and significant weight gains in male WT and Ffar4KO mice (Fig. 1A). In both WT and Ffar4KO mice, these weight gains were due to significant increases in fat mass (Fig. 1B), while lean mass remained unchanged (Fig. 1C), resulting in significant increases in the AI (Fig. 1D), indicating male WT and Ffar4KO mice developed similar levels of obesity. The HFpEF-MetS diet also induced a similar degree of glucose intolerance in response to an intraperitoneal glucose challenge in both male WT and Ffar4KO mice (Fig. 1E, F), suggesting progression toward Type 2 diabetes. Furthermore, the HFpEF-MetS diet produced similar and significant increases in mean arterial pressure in male WT and Ffar4KO mice (Fig. 1G), indicating mild hypertension. Interestingly, the HFpEF-MetS diet induced greater increases in both TG and HDL-C levels in male Ffar4KO mice (Fig. 1H, I). In summary, 20 weeks of the HFpEF-MetS diet produced a similar weight gain, hypertension, and glucose intolerance but increased TG and HDL levels in male Ffar4KO mice relative to WT mice.

### Loss of Ffar4 worsened diastolic dysfunction and microvascular rarefaction induced by MetS in male mice

**Fig. 2:**
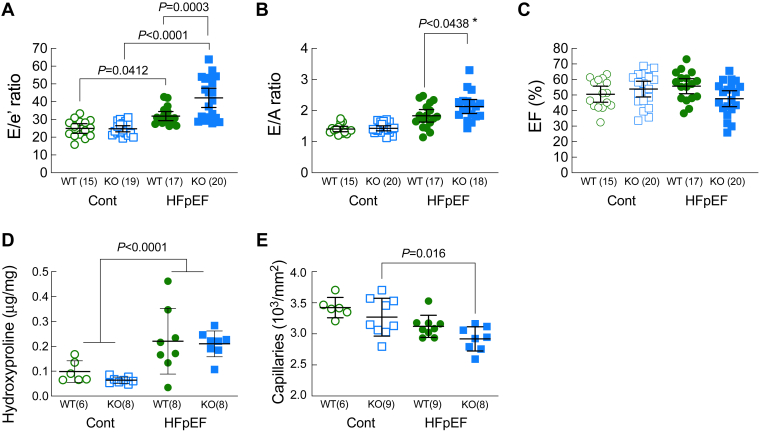
Cardiac function was measured by echocardiography in male WT (green) and Ffar4KO (KO, blue) mice after 20 weeks on the control diet (Cont., open symbols) or HFpEF diet (HFpEF, closed symbols). (A) E/e’ ratio. (B) E/A ratio. (C) Ejection fraction (EF, %). (D) Ventricular fibrosis quantified hydroxyproline content (μg/mg ventricular wet weight). (E) Ventricular myocardial capillary density quantified by Isolectin-B4. Data (A–E) are presented as mean ± 95% CI and were analyzed by two-way ANOVA with Tukey's multiple comparison test. ∗Primary interaction: *P* = 0.0779, Welch’s *t* test for WT HFpEF *versus* Ffar4KO HFpEF, employed due to unequal variance between Control and HFpEF diet groups: *P* = 0.0438. Ffar4, free fatty acid receptor 4; HFpEF, heart failure with preserved ejection fraction.

Previous studies in mice (35, 38) and humans (8) have demonstrated a link between MetS and HFpEF. After 20 weeks, we measured cardiac function by echocardiography in both male WT and Ffar4KO mice to determine if MetS induced HFpEF in WT mice and if loss of Ffar4 worsened cardiac function secondary to MetS (Fig. 2 and supplemental Table 3A). In agreement with previous studies (35, 38), the HFpEF-MetS diet induced significant diastolic dysfunction, evidenced by increased E/e’ ratio and E/A ratios (Fig. 2A, B, green symbols), but ejection fraction was preserved (EF, Fig. 2C, green symbols) in male WT mice. More importantly, in male Ffar4KO mice, the HFpEF-MetS diet significantly worsened diastolic dysfunction relative to male WT mice (E/e’, E/A, Fig. 2A, B, closed green symbols vs. closed blue symbols, supplemental Fig. 1A,B) but again EF was preserved (Fig. 2C).

Two prominent changes to cardiac structure associated with HFpEF are an increase in interstitial fibrosis and microvascular rarefaction (12). After 20 weeks, total cardiac collagen content, measured by hydroxyproline content, was significantly increased to a similar degree in both male WT and Ffar4KO mice (Fig. 2D). Interestingly, there was no detectable change in interstitial fibrosis measured by picrosirius red staining, which stains primarily fibrillar collagens I and III, in either male WT or Ffar4KO mice (supplemental Fig. 2), similar to the very minor changes previously observed in a similar model (35). However, a failure to observe interstitial fibrosis with picrosirius red staining was also noted in a mouse model of chronic kidney disease-induced HFpEF, although a further proteomic analysis revealed profound changes in the extracellular matrix, including many nonfibrillar collagens (39). Furthermore, the HFpEF-MetS diet reduced capillary density in male Ffar4KO mice relative to Ffar4KO mice on the control diet (Fig. 2E and supplemental Fig. 3A). In summary, the loss of Ffar4 in males despite having little to no effect on MetS, significantly worsened diastolic function and microvascular rarefaction (supplemental Fig. 3B), suggesting that Ffar4 is required for an adaptive response to pathological cardiovascular stress induced secondary to MetS in males.

### Loss of Ffar4 decreased GLS, reverse longitudinal peak strain rate, cardiac efficiency, and maximal systolic function in response to MetS in male mice

**Fig. 3:**
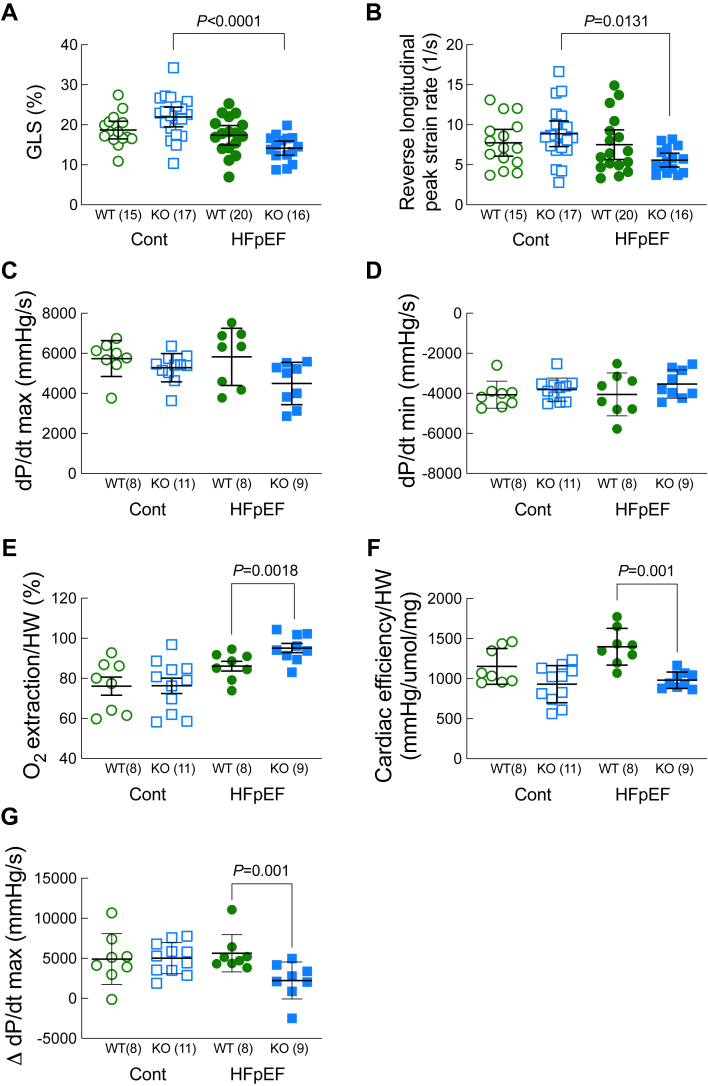
Cardiac function was measured by echocardiographic strain analysis (A, B) or using Langendorff perfused hearts (C–G) from male WT (green) and Ffar4KO (KO, blue) mice after 20 weeks on the control diet (Cont., open symbols) or HFpEF diet (HFpEF, closed symbols). (A) Global longitudinal strain (GLS, %). (B) Reverse longitudinal peak strain rate (1/s). (C) dP/dt max. (D) dP/dt min. (E) O_2_ extraction normalized to heart weight (%). (F) Cardiac efficiency normalized to heart weight (mmHg/μmol/mg). (G) Change in dP/dt from baseline following isoproterenol administration. Data (A–G) are presented as mean ± 95% CI and were analyzed by two-way ANOVA with Tukey's multiple comparison test. Ffar4, free fatty acid receptor 4; HFpEF, heart failure with preserved ejection fraction.

To gain further insight into the effects of MetS on cardiac function, we used echocardiographic strain analysis to assess the effects of the HFpEF-MetS diet on systolic and diastolic function in male WT and Ffar4KO mice. Interestingly, the HFpEF-MetS diet decreased GLS in male Ffar4KO mice (GLS, Fig. 3A), indicating systolic dysfunction in male Ffar4KO mice in response to metabolic syndrome. Although EF was unchanged in either male WT or Ffar4KO mice fed the HFpEF-MetS diet, it is important to note that EF does not reflect myocardial contractility and is load dependent (40, 41). Furthermore, the HFpEF-MetS diet reduced reverse longitudinal peak strain rate in male Ffar4KO mice (Fig. 3B), confirming worsened diastolic function in these mice (42, 43, 44).

To eliminate the confounding effects of vascular loading and neuronal influences on cardiac function, we evaluated cardiac function in male mice using isolated Langendorff-perfused hearts. Surprisingly, in isolated hearts, isometric measures of systolic (dP/dt max) and diastolic function (dP/dt min) were not altered by the HFpEF-MetS diet or loss of Ffar4 (Fig. 3C, D). The absence of diastolic dysfunction during the isometric relaxation of the isolated heart suggests that the worsened diastolic dysfunction observed in male Ffar4 KO hearts is only evident in hearts with dynamic changes in volume that occur in vivo. Further, there were no significant differences in diastolic pressure-volume relationships between male WT and Ffar4KO hearts, a measure of the passive properties of the ventricle across several beats. This implies that a reduction in the rate of cellular relengthening drives the worsened diastolic dysfunction in male Ffar4KO hearts, which is consistent with the decrease in reverse longitudinal peak strain rate observed in these mice. However, we cannot eliminate the possibility that a peripheral factor is influencing the diastolic dysfunction we observed in vivo. Interestingly, the HFpEF-MetS diet increased oxygen extraction from the coronary arteries but reduced cardiac efficiency in male Ffar4KO hearts (Fig. 3E, F), indicating that loss of Ffar4 affects cardiac oxygen delivery and the ability of the heart to convert oxygen into mechanical function and are likely the result of microvascular rarefaction. Finally, while the baseline maximal isometric rate of pressure increase was the same across all groups, the HFpEF-diet attenuated the response to the β-adrenergic receptor agonist isoproterenol (Fig. 3G), indicating that loss of Ffar4 in male mice attenuates maximal systolic function.

### Loss of Ffar4 augmented weight gain without worsening diastolic dysfunction in response to MetS in female mice

**Fig. 4:**
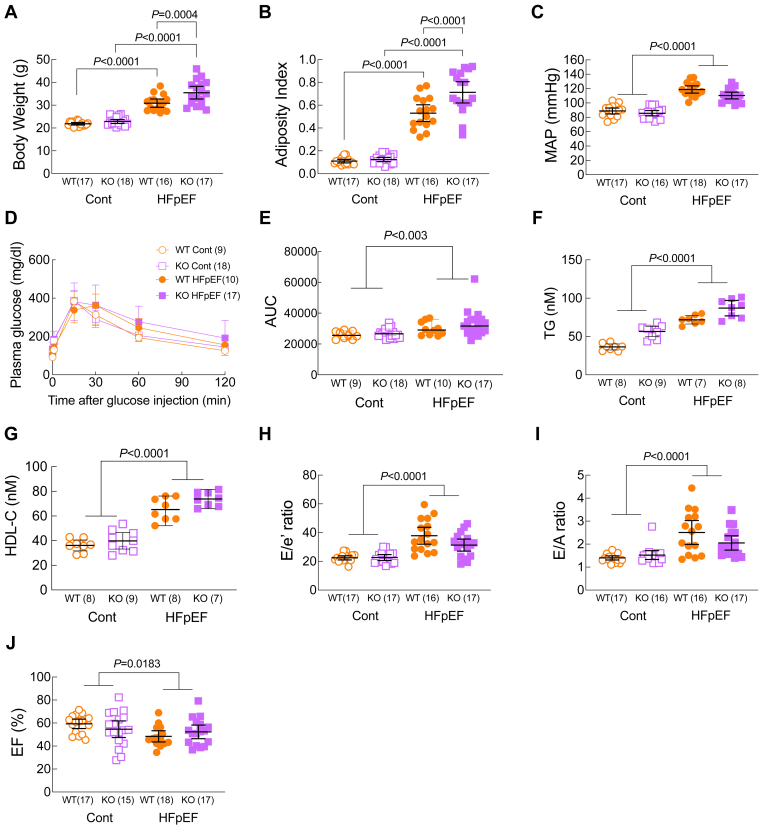
Female WT (orange) and Ffar4KO (KO, purple) mice were fed a control diet (Cont., open symbols) or the combination of a high-fat (42%)/high-sucrose (30%) diet and L-NAME (1 mg/ml) in the drinking water (HFpEF, closed symbols) for 20 weeks. After 20 weeks; (A) body weight was recorded and (B) Adiposity Index (fat mass/lean mass, determined by EchoMRI) was calculated. (C) Mean Arterial Pressure (MAP) measured by tail-cuff. (D) Intraperitoneal glucose tolerance test (IPGTT). (E) calculation of AUC. (F) Triglyceride levels and (G) HDL-C levels were measure from plasma. Cardiac function was measured by echocardiography. (H) E/e’ ratio. (I) E/A ratio. (J) ejection fraction (EF, %). Data (A–I) are presented as mean ± 95% CI and were analyzed by two-way ANOVA with Tukey's multiple comparison test. Ffar4, free fatty acid receptor 4; HFpEF, heart failure with preserved ejection fraction; L-NAME, L-nitroarginine methyl ester.

In female mice, the HFpEF-MetS diet induced significantly greater weight gain in the Ffar4KO mice than WT (Fig. 4A, Summary data: supplemental Table 2B). This additional weight gain in female Ffar4KO mice was due to a significant increase in fat mass, while lean mass remained unchanged, resulting in a significant increase in the AI (Fig. 4B), indicating a greater degree of obesity in the female Ffar4KO mice. However, the HFpEF-MetS diet produced similar degrees of hypertension and similar increases in TG and HDL levels in female WT and Ffar4KO (Fig. 4C, F, G) but interestingly failed to induce glucose intolerance in either WT or Ffar4KO mice (Fig. 4D, E). In female WT mice, the HFpEF-MetS diet induced significant diastolic dysfunction evidenced by increased E/e’ and E/A ratios (Fig. 4H–J, orange symbols, Summary data: supplemental Table 3B). We did observe a small but statistically significant reduction in EF induced by the HFpEF-MetS diet in WT and Ffar4KO female mice (Fig. 4I). However, despite the increased obesity observed in the female Ffar4KO mice, the HFpEF-MetS diet did not result in any further worsening of diastolic function relative to female WT mice (Fig. 4H–J, closed orange vs. closed purple symbols). In summary, the loss of Ffar4 in females resulted in greater obesity in response to the HFpEF-MetS diet but this had no worsening effect on cardiac function as was observed in male Ffar4KO mice. This sex-based difference was similar to our prior report (34), and we did not investigate the cardiac phenotype in females further.

### Loss of Ffar4 increased the 12-HETE/18-HEPE ratio in HDL in response to MetS in males but not females, suggesting a systemic proinflammatory state in Ffar4KO males

We previously demonstrated that in cardiac myocytes, Ffar4-cPLA2α signaling specifically induced the production of the EPA-derived, proresolving oxylipin 18-HEPE, which protected cardiac myocytes from oxidative stress (34). Interestingly, a recent report indicated that the AA-derived, proinflammatory oxylipin 12-HETE exacerbates endothelial dysfunction and is associated with the development of HFpEF, and that antagonizing 12-HETE improved outcomes in mouse model of HFpEF in Type 2 diabetic db/db mice (45). Therefore, we investigated how loss of Ffar4 in the context of HFpEF secondary to MetS might impact 18-HEPE and 12-HETE levels. Initially, we measured 18-HEPE and 12-HETE levels in circulating HDL as an index of systemic Ffar4-cPLA2α activity.

#### 18-hydroxyeicosapentaenoic acid

**Fig. 5:**
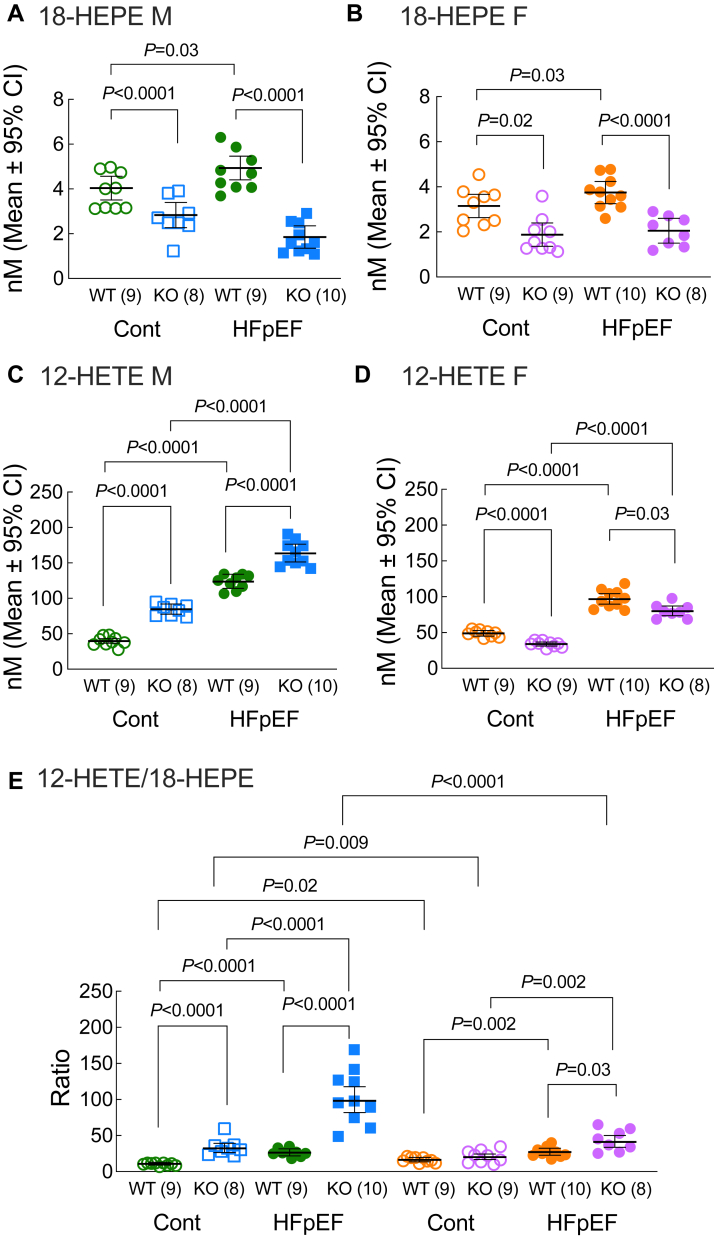
After 20 weeks on diet (Cont., open symbols, HFpEF, closed symbols), plasma was collected from both male and female, WT and Ffar4KO mice (Male: WT, green; KO, blue, Female: WT, orange; KO, purple) and HDL oxylipin content was detected by liquid chromatography/mass spectrometry. (A, C) Levels of the 18-HEPE, an EPA-derived, proresolving oxylipin and 12-HETE, an AA-derived, proinflammatory oxylipin in HDL from males. (B, D) Levels of 18-HEPE and 12-HETE in HDL from females. (E) 12-HETE/18-HEPE ratio in HDL from males and females. Data are presented as mean ± 95% CI and were analyzed by two-way ANOVA with Tukey's multiple comparison test. 18-HEPE, 18-hydroxyeicosapentaenoic acid; 12-HETE, 12-hydroxyeicosatetraenoic acid; Ffar4, free fatty acid receptor 4; HFpEF, heart failure with preserved ejection fraction.

In WT mice, the HFpEF-MetS diet slightly increased 18-HEPE levels in HDL from both males (Fig. 5A, green symbols) and females (Fig. 5B, orange symbols). In Ffar4KO male and female mice, 18-HEPE levels were lower in HDL from mice on the control diet (Fig. 5A, B, open symbols), while the HFpEF-MetS diet tended to reduce 18-HEPE levels in HDL from males (Fig. 5A, blue symbols, Fig. 5B, purple symbols). In total, loss of Ffar4 significantly reduced basal levels of 18-HEPE in HDL and prevented any compensatory increase in 18-HEPE (Fig. 5A, B, closed symbols).

#### 12-hydroxyeicosatetraenoic acid

In WT mice, the HFpEF-MetS diet increased 12-HETE levels in HDL from male (Fig. 5C, green symbols) and female mice (Fig. 5D, orange symbols). Conversely, in Ffar4KO mice, 12-HETE levels were elevated in HDL from males (Fig. 5C, open symbols) but lower in in females (Fig. 5D, compare open symbols). However, the HFpEF diet increased 12-HETE levels in HDL from both males (Fig. 5C, blue symbols) and females (Fig. 5D, purple symbols). In total, loss of Ffar4 had a divergent effect on basal levels of 12-HETE in HDL from males (higher) and females (lower). While the HFpEF-MetS diet increased 12-HETE in HDL of both sexes, this basal difference accounted for the higher absolute levels of 12-HETE in Ffar4KO males relative to WT (Fig. 5C, closed symbols) and lower absolute levels in females (Fig. 5D, closed symbols).

#### 12-HETE/18-HEPE ratio

We subsequently calculated the 12-HETE (proinflammatory) to 18-HEPE (proresolving) ratio as an index of the systemic inflammatory state. In WT mice, the HFpEF-MetS diet slightly increased the 12-HETE/18-HEPE ratio in males (Fig. 5E, green symbols) and females (Fig. 5E, orange symbols). In Ffar4KO mice, the basal 12-HETE/18-HEPE ratio was elevated in males relative to females (Fig. 5E, open symbols), owing largely to elevated basal levels of 12-HETE in males. However, the HFpEF-MetS diet provoked a dramatic increase in the 12-HETE/18-HEPE ratio in the Ffar4KO males, but not Ffar4KO females (Fig. 5E, blue vs. purple closed symbols), largely due to the combination of decreased 18-HEPE levels and increased 12-HETE levels in male Ffar4KO mice compared to female KO mice.

In summary, these results reveal some generalized trends in systemic Ffar4-cPLA2α–mediated oxylipin levels. First, the loss of Ffar4 was correlated with decreased levels of 18-HEPE but increased levels of 12-HETE in males but not females. Second, in WT mice, MetS was associated with increased 18-HEPE and 12-HETE levels, such that the overall balance between the proinflammatory 12-HETE and proresolving 18-HEPE was largely maintained. Finally, and most importantly, the loss of Ffar4 dramatically impaired the ability to increase 18-HEPE levels while further increasing 12-HETE levels in males.

### Loss of Ffar4 increased the basal 12-HETE/18-HEPE ratio in the heart and in response to MetS in males, suggesting a proinflammatory state in male Ffar4KO hearts

Following on the findings that metabolic syndrome increased the 12-HETE/18-HEPE ratio in HDL from male Ffar4KO mice, we measured 18-HEPE and 12-HETE levels in both the esterified (membrane) and nonesterified (NEOx, free) fractions from male hearts to understand how the loss of Ffar4 in the context of HFpEF secondary to MetS affected Ffar4-cPLA2α activity in the male heart specifically.

#### 18-hydroxyeicosapentaenoic acid

**Fig. 6:**
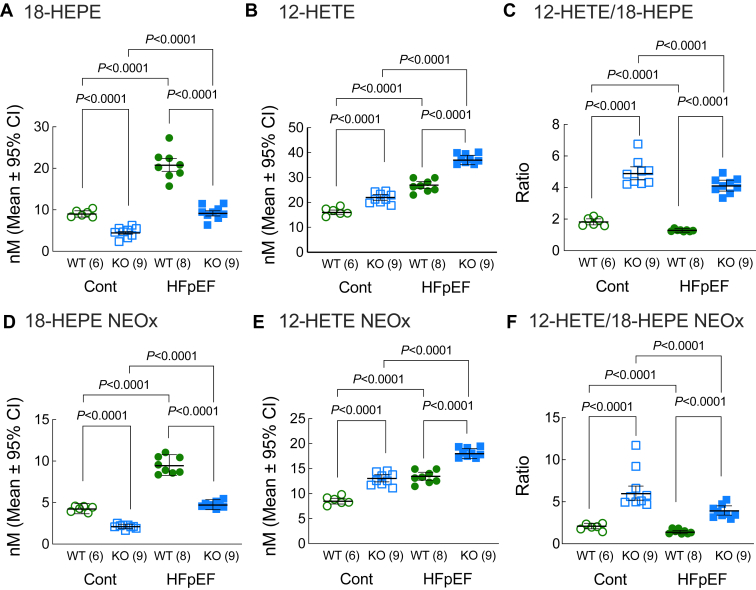
After 20 weeks on diet (Cont., open symbols, HFpEF, closed symbols), hearts were harvested from both male, WT (WT, green) and Ffar4KO (KO, blue) mice, and heart oxylipin content in the esterified and nonesterified (NEOx) fractions was detected by liquid chromatography/mass spectrometry. (A, D) 18-HEPE, esterified, and non-esterified. (B, E): 12-HETE, esterified, and nonesterified. (C, F) 12-HETE/18-HEPE ratio, esterified, and nonesterified. Data are presented as mean ± 95% CI and were analyzed by two-way ANOVA with Tukey's multiple comparison test. 18-HEPE, 18-hydroxyeicosapentaenoic acid; 12-HETE, 12-hydroxyeicosatetraenoic acid; Ffar4, free fatty acid receptor 4; HFpEF, heart failure with preserved ejection fraction.

Similar to HDL, 18-HEPE levels were lower in the male Ffar4KO heart at baseline (Fig. 6A, D, open symbols). In male WT hearts, the HFpEF-MetS diet increased 18-HEPE levels heart 2-fold (Fig. 6A, D, green symbols), while in the Ffar4KO, 18-HEPE was also increased almost 2-fold, but the absolute levels of 18-HEPE in the Ffar4KO were much lower (Fig. 6A, D, blue symbols).

##### 12-hydroxyeicosatetraenoic acid

12-HETE levels in the heart mirrored changes observed in HDL. Basal 12-HETE levels were higher in the Ffar4KO (Fig. 6B, E, open symbols), and the HFpEF-MetS diet induced similar increases in the 12-HETE in WT (Fig. 6B, E, green symbols) and Ffar4KO hearts (Fig. 6B, E, blue symbols). This resulted in high levels of 12-HETE in the Ffar4KO heart relative to WT (Fig. 6B, E, closed symbols).

##### 12-HETE/18-HEPE ratio

Interestingly, the 12-HETE/18-HEPE ratio was increased in Ffar4KO hearts both at baseline and in response to the HFpEF-MetS diet (Fig. 6C, F), suggesting a proinflammatory state in the Ffar4KO heart even at baseline.

### Expression of Ffar4 and ChemR23 (*Cmklr1*), a receptor for E-series resolvins, was increased in male HFpEF hearts

**Fig. 7:**
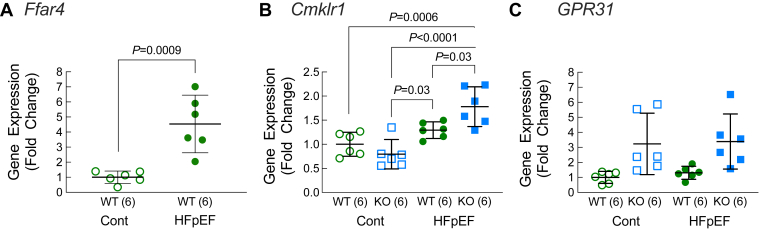
A–C: After 20 weeks on diet (Cont., open symbols, HFpEF, closed symbols), RNA was isolated from male WT (WT, green) and Ffar4KO (KO, blue) hearts, and (A) Ffar4 (*Ffar4*), (B) ChemR23 (*Cmklr1*) , and (C) GPR31 (*Gpr31*) mRNA levels were quantified by qRT-PCR. Data are presented as mean ± 95% CI and were analyzed by two-way ANOVA with Tukey's multiple comparison test. Ffar4, free fatty acid receptor 4; HFpEF, heart failure with preserved ejection fraction.

18-HEPE and 12-HETE are agonists that can lead to downstream activation of GPCR signaling, 18-HEPE through conversion to E-Series resolvins (RvE1) and activation of ChemR23 (*Cmklr1*) (46) and 12-HETE through activation of GPR31 (*Gpr31*) (47) and the leukotriene B4 receptor (48). Therefore, to define the relationship between the increased levels of 18-HEPE, decreased levels of 12-HETE in Ffar4KO mice, and expression of their cognate receptors in the heart, we measured whole heart *Ffar4*, *Cmklr1*, and *Gpr31* mRNA expression. In WT mice, the HFpEF-MetS diet significantly increased Ffar4 expression (Fig. 7A), a somewhat surprising finding given that we previously found that Ffar4 expression is decreased in human heart failure (34). In Ffar4KO mice, the HFpEF-MetS diet significantly increased ChemR23 expression relative to WT (Fig. 7B), while GPR31 expression was elevated in the Ffar4KO heart regardless of diet (Fig. 7C). The increased levels of ChemR23 and GPR31 in the male Ffar4KO heart, along with the changes in their respective ligands support the idea that the HFpEF-MetS diet might have induced a more proinflammatory state in the male Ffar4KO heart.

### Loss of Ffar4 increased CD64^+^ macrophages in response to MetS in male hearts, which correlated with worsened ventricular remodeling

**Fig. 8:**
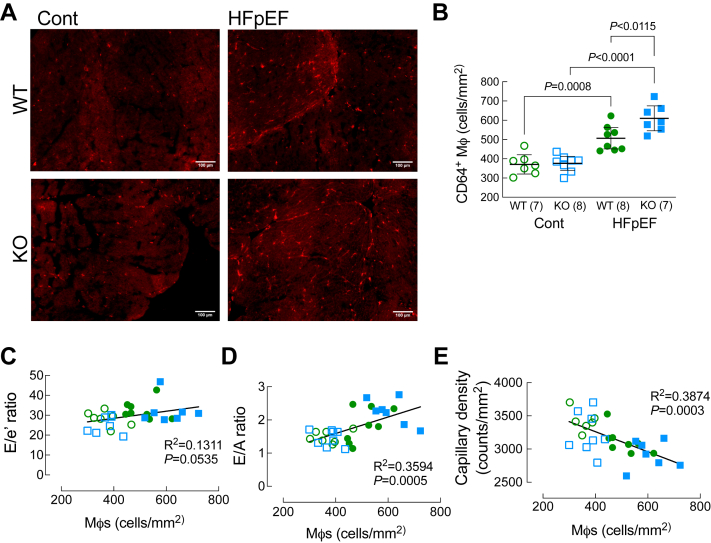
(A, B) After 20 weeks on diet (Cont., open symbols, HFpEF, closed symbols), ventricular sections from male WT (WT, green) and Ffar4KO (KO, blue) hearts were stained CD64 to detect the total macrophage population (A) and total CD64^+^ cells were counted (B). Data (B) are presented as mean ± 95% CI and were analyzed by two-way ANOVA with Tukey's multiple comparison test. (C–E) Total CD64^+^ macrophages were correlated with markers of HFpEF remodeling: (C) E/e’ ratio; (D) E/A ratio; and (E) Capillary density. Ffar4, free fatty acid receptor 4; HFpEF, heart failure with preserved ejection fraction.

Recently, it has become clear that cardiac macrophages represent a diverse cell population that plays a critical role in cardiac homeostasis and the response to cardiac injury (Reviews: (49, 50)). Furthermore, systemic inflammation and recruitment of macrophages to the heart might promote HFpEF, and a few studies have suggested that macrophage numbers are increased in models of hypertension/chronic kidney disease or aging that resemble HFpEF (51, 52, 53), as well as human HFpEF patients (53). With the increase in the 12-HETE/18-HEPE ratio in the male Ffar4KO mice suggesting a more inflammatory state in the heart, we hypothesized that macrophage numbers would be increased in Ffar4KO hearts. To test this hypothesis, we quantified the number of CD64^+^ macrophages in male WT and Ffar4KO hearts. In male WT hearts, the HFpEF-MetS diet increased the number of CD64^+^ macrophages, indicating an increase in total macrophage number, while the HFpEF-MetS diet induced a further significant increase in CD64^+^ macrophages in the male Ffar4KO hearts (Fig. 8A, B). The increase CD64^+^ macrophages in WT hearts in response to MetS supports the hypothesis that inflammation drives HFpEF remodeling. More importantly, the further increase in this CD64^+^ macrophage population in Ffar4KO hearts in response to MetS is consistent with the increased 12-HETE/18-HEPE ratio in these mice. Interestingly, the number of CD64^+^ macrophages was positively correlated with diastolic dysfunction (E/e’, Fig. 8C, E/A, Fig. 8D) and inversely correlated with microvascular rarefaction (Fig. 8E), again supporting the hypothesis that inflammation drives HFpEF remodeling.

## Discussion

Of the roughly 6 million total cases of heart failure in the US, the prevalence of HFpEF now exceeds 50% (54), and despite the recent success of empagliflozin in EMPEROR-Preserved (4), therapeutic options for HFpEF remain limited. Here, we have identified a novel cardioprotective role for Ffar4 in the context of HFpEF secondary to MetS, suggesting a potentially new therapeutic target for the management of cardiometabolic disease. In response to a dietary challenge designed to induce MetS, modified from the original 2-hit HFpEF model proposed by Schiattarella *et al.* (35), we found that systemic deletion of Ffar4 surprisingly did not affect development of MetS but significantly worsened ventricular remodeling in male mice. Conversely, in female mice lacking Ffar4, this HFpEF-MetS diet induced greater weight gain but no worsening of ventricular remodeling.

Loss of Ffar4 decreased levels of the EPA-derived, proresolving oxylipin 18-HEPE, while increasing the levels of the AA-derived, proinflammatory oxylipin 12-HETE in HDL and hearts from male mice to a far greater degree than from female mice fed the HFpEF-Mets diet. This resulted in a dramatic increase in the 12-HETE/18-HEPE ratio in male Ffar4KO mice fed the HFpEF-Mets diet, which suggests the following: *1*) In males, loss of Ffar4 induced a potentially more proinflammatory state both systemically and in the heart in response to MetS that correlated with the worsened remodeling in males and *2*) In females, loss of Ffar4 did not increase the 12-HETE-/18-HEPE ratio in response to MetS, largely due to lower basal levels of 12-HETE in female Ffar4KO mice relative to males, potentially providing a plausible mechanistic explanation for the observed sex-based difference.

18-HEPE is the sole precursor for RvE, which are ligands for the GPCR ChemR23 (*Cmklr1*)) (46), whereas 12-HETE is a ligand for GPR31 (*Gpr31*) (47) and leukotriene B4 receptor (*Blt2*) (48). In male Ffar4KO mice, ChemR23 expression was lower at baseline, but showed a much larger increase in response to the HFpEF-MetS diet, while GPR31 was elevated regardless of diet in the male Ffar4KO, suggesting a potential link between decreased 18-HEPE levels, increased 12-HETE levels, and macrophage function in male Ffar4KO mice on the HFpEF-MetS diet. As mentioned, HFpEF is proposed to be a disease of systemic inflammation (1), and in fact, we provide the first evidence in this 2-hit HFpEF-MetS model that CD64^+^ macrophage numbers are increased in WT hearts. More importantly, we observed a further increase in CD64^+^ macrophages in male Ffar4KO hearts, correlating with the heightened proinflammatory state in these mice. Briefly, the data suggest that Ffar4 controls the proinflammatory/anti-inflammatory oxylipin balance in the heart to modulate macrophage function and attenuate HFpEF remodeling, but more work will be needed to define the relationship between Ffar4-mediated regulation of oxylipin levels and immune cell function.

Inflammation secondary to comorbidities associated with HFpEF, including MetS and chronic kidney disease, are proposed to drive HFpEF remodeling (1, 6, 7). In support of this hypothesis, inflammation associated with hypertension, obesity, and diabetes predicted incident HFpEF, but not HFrEF in the Health, Aging, and Body Composition study (Health ABC) (55). Plasma markers of inflammation including soluble IL-1 receptor-like 1, C-reactive protein (CRP), and growth and differentiation factor 15 are high in HFpEF patients (56, 57). Furthermore, CRP is correlated with LV end-DP and CRP is associated with asymptomatic diastolic dysfunction in MetS (58). In patients with HFpEF, LV biopsies showed evidence of increased VCAM expression, increased CD3^+^, CD11^+^, and CD45^+^ leukocytes, and increased TGFβ1 expression, suggesting a link between myocardial inflammation and HFpEF (59). In the heart, inflammation can induce endothelial cell death and dysfunction leading to microvascular rarefaction and disruption of endothelial-myocyte communication with decreased NO resulting in stiff, hypertrophied cardiac myocytes (60), as well as recruitment of inflammatory monocytes and macrophages that can induce interstitial fibrosis (59, 60). Here, we found an increased number of CD64^+^ macrophages in male WT hearts on the HFpEF-MetS diet, with a further increase in CD64^+^ macrophages in male Ffar4KO hearts, supporting the assertion that inflammation drives HFpEF. However, it remains to be determined if the increased number of CD64^+^ macrophages observed in WT hearts and the even greater increase observed in Ffar4KO hearts was causative to the observed worsened ventricular remodeling in response to MetS.

Here, we also report the first data demonstrating that loss of Ffar4 reduced cardiac 18-HEPE levels in vivo, supporting our prior in vitro data from cardiac myocytes indicating that activation of Ffar4 increased 18-HEPE levels (34). Furthermore, loss of Ffar4 increased 12-HETE levels, increasing the 12-HETE/18-HEPE ratio, and suggesting a more pro-inflammatory state in the male Ffar4KO heart both at baseline and in response to the HFpEF-MetS diet. In the heart, prior studies indicate that 12-HETE worsens ischemic injury, induces maladaptive hypertrophy, and worsens HF (61), and a recent study indicated that 12-HETE specifically worsens HFpEF remodeling (45). Ultimately, the increased 12-HETE/18-HEPE ratio in male Ffar4KO hearts was associated with increased CD64^+^ macrophage numbers, which correlated with worsened HFpEF remodeling. This suggests a novel paradigm in which one GPCR, Ffar4, functions in a feed-forward mechanism to regulate downstream GPCR agonists (18-HEPE, ChemR23 (*Cmkrl1*); 12-HETE, GPR31 (*Gpr31*)) to attenuate the cardiac inflammatory response to cardiometabolic disease.

Recently, we demonstrated that in adult cardiac myocytes, Ffar4-cPLA2α signaling directly and uniquely increased synthesis of 18-HEPE (34), which helps to explain the reduced levels of 18-HEPE in HDL and hearts of Ffar4KO mice both at baseline and in response to the HFpEF-MetS diet. Increased levels of 18-HEPE, secondary to EPA-supplementation or in fat-1-transgenic mice that convert ω6- to ω3-PUFAs, are associated with prevention of atherosclerosis and pathologic ventricular remodeling post-TAC (62, 63). Furthermore, 18-HEPE directly inhibits cardiac myocyte death (34), and exogenous, unesterified 18-HEPE prevents post-TAC remodeling (63). Collectively, these findings suggest that 18-HEPE is cardioprotective but its mechanism of action remains unclear. To date, no receptor has been identified for 18-HEPE, however, 18-HEPE is the precursor for RvE, which signal through ChemR23 (*Cmklr1*). ChemR23 is expressed in macrophages (64), smooth muscle cells (62), endothelial cells (65), and adipocytes (66), and based on direct effects of 18-HEPE to prevent cardiac myocyte cell death (34) and RvE1 infusion to attenuate postinfarction remodeling (67), we speculate that ChemR23 might also be expressed in cardiac myocytess. Interestingly, ChemR23 binds to two entirely different ligands; the peptide chemerin, a macrophage chemoattractant (68), and RvE1 that are derived from EPA/18-HEPE (46). In macrophages, ChemR23 expression is restricted to naïve and more proinflammatory macrophages, which respond to chemerin produced in inflamed tissue to recruit macrophages, while RvE1 promotes a shift in proinflammatory macrophages toward a more proresolving phenotype (69). In a mouse atherosclerosis model, EPA supplementation increased 18-HEPE levels and prevented atherosclerosis progression. However, systemic deletion of ChemR23 in this context also increased macrophage uptake of oxidized LDL, reduced phagocytosis, and increased plaque size (62). However, a separate study seemed to reach the opposite conclusion (70), which might reflect differences in the balance of the two ChemR23 ligands. In total, the reduced levels of 18-HEPE in male Ffar4KO mice suggests the potential for more proinflammatory signaling through macrophage ChemR23.

Positive results with icosapent ethyl (EPA) in the Reduction of Cardiovascular Events with EPA-Intervention Trial (REDUCE-IT) (71) and the Effect of Vascepa in Improving Coronary Atherosclerosis in People with High TGs Taking Statin Therapy Trial (EVAPORATE) (72) to improve cardiovascular outcomes have renewed interest in the mechanistic basis for EPA-mediated cardioprotection. Following the original identification of Ffar4 (GPR120) as a receptor for long-chain FAs (15), there has been considerable interest in the idea that Ffar4 mediates these protective effects. However, detailed in vitro studies of Ffar4 pharmacology suggest that in general, PUFAs, including ω3-PUFAs (EPA) and ω6-PUFAs (AA), have relatively similar efficacy and potency and are not biased agonists (13). Assuming this is correct, it presents a conundrum in explaining the beneficial effects of ω3-PUFAs versus ω6-PUFAs in terms of Ffar4 ligand binding or activation of immediate downstream signaling pathways. Adding to this complexity is the idea that ω3-PUFAs and EPA in particular have several proposed mechanisms of action including receptor-mediated signaling through Ffar1/4 and peroxisome proliferator-activated receptors, but also production of oxylipins (e.g., 18-HEPE), and direct effects on membrane structure (73). Our current results and previous work (34) indicate that Ffar4-cPLA2α signaling in cardiac myocytes shows surprising specificity to induce the production of 18-HEPE. Given that cPLA2α does not have a reading function and will cleave whichever PUFA is found in the sn-2 position of membrane phospholipids, it is logical to suggest that altering ω3/ω6 levels in membrane phospholipids through dietary supplementation could alter production of their cognate oxylipins. This concept is also consistent with our assertion that the mechanism by which Ffar4 prevents HFpEF remodeling is by controlling proinflammatory/anti-inflammatory oxylipin balance to attenuate inflammation.

In female Ffar4KO mice, the HFpEF-Mets diet induced a greater accumulation of fat but surprisingly, this did not translate to worsened cardiac outcomes. If anything, female Ffar4KO hearts showed a trend toward less diastolic dysfunction. Although there is some controversy, more studies seem to suggest that loss of Ffar4 worsens metabolic outcomes with little effect on weight gain in mice challenged with HFD (20, 21, 22, 23). Interestingly, none of these prior studies examined females. Furthermore, we specifically assessed cardiometabolic disease, by including a hypertensive challenge with the HFD, while none of these prior studies measured cardiac outcomes. Nonetheless, our results indicate that loss of Ffar4 induced 15% more weight gain over 20 weeks in female mice, suggesting that Ffar4 attenuates obesity in females. This finding might inform previous studies in which the examination of the Ffar4 R270H polymorphism and obesity in humans has produced conflicting results (21, 26). Additionally, in the context of human disease, HFpEF patients tend to be older and female (2), a population in which all HF is more prevalent (54). In our HFpEF-MetS model, female WT mice developed slightly less diastolic dysfunction, similar to previous reports suggesting female mice are protected from HFpEF (74), a seeming discrepancy with humans. Further, despite the increased weight gain, female Ffar4KO mice showed no worsening of diastolic dysfunction relative to female WT mice. One possible explanation for the sex-based difference between humans and mice is that this might simply reflect a higher survival rate of human females with cardiovascular disease who become more susceptible to HFpEF with age.

Interestingly, we observed a significant sex-based difference in the 12-HETE/18-HEPE ratio in HDL, with a higher ratio in the male versus female Ffar4 KO mice that was associated with worse outcomes in the males. Interestingly, 18-HEPE levels were roughly equivalent between male and female mice in each diet group, despite lower 18-HEPE levels in the Ffar4KO mice relative to WT mice. As noted already, 18-HEPE is cardioprotective, and reduced 18-HEPE levels likely contribute to worsening remodeling in the males but do not explain the sex-based difference. Conversely, there were significant differences in 12-HETE based on sex, genotype, and diet. Furthermore, 12-HETE was elevated in male Ffar4KO mice at baseline and increased on the HFpEF-MetS diet. Conversely, in female Ffar4KO mice, 12-HETE levels were much lower at baseline, and while increased on the HFpEF-Mets diet, the absolute levels were much lower than the male Ffar4KO mice on the HFpEF-MetS diet. In humans, 12-HETE levels are elevated in subjects with type 2 diabetes and diastolic dysfunction (45). Additionally, 12-HETE levels are elevated in db/db mice that develop a HFpEF-like phenotype, and blocking 12-HETE attenuated the HFpEF phenotype (45). Furthermore, in mice fed a high-sucrose diet, 5-HETE, and 12-HETE were significantly downregulated in female mice versus males (75). Therefore, 12-HETE might play and important role in mediating the sex-based differences observed in the current study. However, future studies will have to examine oxylipin levels in male and female hearts to confirm this hypothesis.

In conclusion, we demonstrate for the first time that Ffar4 attenuates cardiometabolic disease and present novel association between Ffar4 modulation of oxylipin levels, immune cell populations in the heart, and prevention of HFpEF remodeling. Furthermore, we suggest a plausible mechanism through Ffar4-cPLA2α–mediated EPA-derived oxylipin synthesis that sheds new light on the basis of EPA-mediated cardioprotection. Finally, based on the experimental success of synthetic Ffar4 agonist to attenuate metabolic disease (27, 28, 29), we suggest that Ffar4 might be a novel therapeutic target in cardiometabolic disease. However, more work is required to define the underlying mechanisms for the Ffar4-mediated cardioprotective effects and the basis for the difference between the sexes.

## Data availability

All data for this manuscript are included in the manuscript.

## Supplemental data

This article contains supplemental data.

## Conflict of interest

The authors declare that they have no conflicts of interest with the contents of this article.
